# Risk Factors for Developing Venous Thromboembolism in Patients With Advanced ALK-Rearranged NSCLC

**DOI:** 10.1016/j.jtocrr.2026.101003

**Published:** 2026-04-23

**Authors:** Caroline Kamali, Georgios Tsakonas, Rolf Lewensohn, Lena Kanter, Hanna Vikman, Anders Berglund, Elena Nedbaylo, Per Hydbring, Kristina Viktorsson, Luigi De Petris, Simon Ekman

**Affiliations:** a Department of Oncology-Pathology, Karolinska Institutet, Stockholm, Sweden; b Theme Cancer, Medical Unit Head and Neck, Lung and Skin Tumors, Thoracic Oncology Center, Karolinska University Hospital, Stockholm, Sweden; c EpiStat AB, Uppsala, Sweden

**Keywords:** Non–small cell lung cancer, Anaplastic lymphoma kinase translocation, *EML4-ALK* fusion variants, Venous thromboembolism, Advanced disease

## Abstract

**Introduction:**

Patients with NSCLC have an increased risk of developing venous thromboembolism (VTE), a condition associated with a poorer prognosis. Those with *ALK* rearrangements have a three- to five-fold higher risk of VTE than those with other oncogenic drivers. This study aimed to investigate the incidence, risk factors, and impact of VTE on overall survival.

**Methods:**

We retrospectively analyzed patients with advanced ALK-positive NSCLC treated at Karolinska University Hospital from 2009 to 2021. VTE events were recorded from 90 days before histologic cancer diagnosis to death or end of follow-up. The impact of VTE on survival was assessed using a time-dependent Cox model, and risk factors were evaluated using Fine-Gray competing risk regression.

**Results:**

A total of 97 patients were included, of whom 35 (36.1%) developed VTE. Pulmonary embolism accounted for 24 cases (68.6%), with 16 events (45.7%) occurring more than 6 months after treatment initiation. The median time from diagnosis to the first clot was 167 days. Patients with VTE had a 3.43-fold increased risk of death. Competing risk regression analysis revealed that the presence of baseline adrenal metastasis (hazard ratio [HR] = 2.92, 95% confidence interval [CI]: 1.19–7.16), leukocyte count more than 11 × 10^9^/L (HR = 3.74, 95% CI: 1.91–7.31), and hemoglobin less than 10 g/dL (HR = 5.18, 95% CI: 2.07–12.94) significantly increased VTE risk, whereas albumin more than or equal to 3.5 g/dL reduced the risk (HR = 0.24, 95% CI: 0.10–0.56).

**Conclusion:**

In advanced ALK-positive NSCLC, baseline adrenal metastases, leukocytosis, and anemia were associated with increased VTE risk and may warrant heightened clinical vigilance.

## Introduction

Venous thromboembolic events (VTEs) are a common complication in patients with cancer and represent the third most prevalent acute cardiovascular condition after myocardial infarction and stroke.^1^^,^^2^

The incidence of VTE is particularly high among patients with NSCLC, and its presence is associated with a poorer prognosis than in those without VTE.^3^, ^4^, ^5^, ^6^, ^7^ Approximately 8% to 15% of all patients with NSCLC experience thrombosis during their disease course, with the highest risk observed in those with adenocarcinoma subtype.^8^^,^^9^

One targetable oncogenic driver in NSCLC adenocarcinoma is chromosomal rearrangements of the *ALK* gene.^10^ Previous studies have indicated that the incidence of VTE in patients with ALK-rearranged disease may be three up to five times higher compared with those whose tumors express other oncogenic drivers.^8^ This increased risk may be related to abundant mucin production, a known prothrombotic factor, in patients with ALK-positive NSCLC; however, the underlying biological mechanisms remain unclear.^11^

Furthermore, some studies suggest that *ALK* rearrangements may induce tissue factor (TF) expression, which could play a central role in tumor-associated hypercoagulability.^12^, ^13^, ^14^, ^15^, ^16^ Therefore, this patient group might benefit from prophylactic anticoagulant therapy from the time of lung cancer (LC) diagnosis.

More than 90 different *ALK* fusion partners have been identified, with *EML4* being the most common, accounting for approximately 80% of all *ALK* fusions.^17^ All these variants retain the entire ALK intracellular kinase domain but differ in their breakpoints and in the fusion with the *EML4* gene. The three most common variants 1, 2, and 3a/b are expressed in 33%, 10%, and 29% of EML4-ALK positive cases, respectively. Most variants, except for 3a/b and 5a/b, contain the tandem atypical propeller (TAPE) domain in the EML protein, though in varying proportions, which may contribute to the different biological properties found among the diverse fusion variants.^18^, ^19^, ^20^

The first-line therapy for managing patients diagnosed with having advanced ALK-positive NSCLC is the utilization of targeted therapy with tyrosine kinase inhibitors (TKIs), replacing the formerly established standard of care, which primarily involved conventional systemic chemotherapy, due to enhanced response rates and superior clinical outcomes.^21^, ^22^, ^23^ In this new era of oncogenic driver–directed therapy, the risk of VTE in LC remains largely unexplored. In the context of the increasingly effective therapies and extended overall survival (OS) with the introduction of ALK TKIs, evaluating risk factors for thromboembolic events is clinically crucial as these events can worsen prognosis. Such an evaluation will help physicians identify patients who might benefit from anticoagulant prophylaxis.

The Khorana Risk Score (KRS) is used for assessing VTE risk.^24^ It incorporates the following five clinical and laboratory risk factors: cancer type, platelet count, hemoglobin level (including erythropoiesis-stimulating agent use), white blood cell count, and body mass index. Within this scoring system, LC is classified as a high-risk malignancy. Although the KRS is designed to assess the risk of VTE and guide thromboprophylaxis in patients with cancer, its accuracy appears less reliable, particularly in LC populations.^25^^,^^26^ Importantly, the predictive value of KRS for VTE in patients with ALK-positive NSCLC remains uncertain, warranting further studies to evaluate its utility.

In a previous study,^27^ we analyzed the clinical characteristics and treatment outcomes of patients with ALK-positive NSCLC treated at Karolinska University Hospital. The primary objective of this study, in contrast, was to investigate the association between VTE incidence and different *ALK* fusion variants, including OS in a cohort of patients with ALK-positive NSCLC treated at Karolinska University Hospital in Stockholm, Sweden. In addition, our aim was to identify risk factors for VTE and to validate KRS in the context of ALK-driven NSCLC.

## Materials and Methods

### Study Population

This was a retrospective cohort study conducted using electronic health records from the Karolinska University Hospital in Stockholm. The study included all patients diagnosed with ALK-rearranged NSCLC who were treated between January 2009 and December 2021. Only patients with unresectable or not amenable to definitive radiotherapy stage III or stage IV disease were included, whereas those with early stage disease, a documented history of VTE occurring more than 90 days before the NSCLC diagnosis, ongoing treatment with continuous therapeutic doses of anticoagulants, or an inherited clotting disorder were excluded.

The occurrence of VTE was documented from 90 days before the histologic diagnosis of cancer to death or the end of the follow-up period. If a patient experienced both a pulmonary embolism (PE) and a simultaneous deep venous thrombosis (DVT) of the lower extremities, or if having recurrent VTEs, these events were counted only once and classified collectively as VTE. Both symptomatic and incidental VTEs, confirmed by imaging studies, were included in the analysis.

### Data Collection

The study was approved by the Regional Ethical Review Board in Stockholm (Dnr 2022-00323-01). Informed consent was waived due to the study's retrospective design. The data were collected using patients’ electronic medical records and pseudonymized using identification codes. The following variables were retrospectively collected: demographic data including age and sex; smoking status at diagnosis; Eastern Cooperative Oncology Group performance status; comorbidities; body mass index; complete blood cell count; tumor stage according to the eighth edition of the TNM classification; metastatic sites; oncologic treatment; histopathology; thromboembolic events; location of VTE; clinical presentation of VTE; hospitalization and/or surgery; and death or last follow-up. All laboratory parameters were collected at the time of NSCLC diagnosis, before initiation of systemic treatment, and were used as baseline laboratory values. The International Classification of Diseases, Tenth Revision, codes were used to diagnose VTE and comorbidities. KRS was calculated for each patient according to the algorithm.

### Detection of ALK Status and Fusion Variants

*ALK* rearrangements were detected using the following validated clinical routine methods: fluorescent in situ hybridization, immunohistochemistry,^28^ reverse transcription polymerase chain reaction (RT-PCR),^29^ and next-generation sequencing (NGS).^30^ Immunohistochemistry was performed on formalin-fixed, paraffin-embedded tissue using the ALK/p80 rabbit monoclonal antibody (clone SP8) on the Leica BOND-II platform. Confirmation of *ALK* fusion and variant typing was conducted using RNA-based NGS on the Ion GeneStudio S5 system with the Ion Torrent Oncomine Childhood Cancer Research Assay.

### Statistical Analysis

Descriptive statistics were used to summarize both categorical and continuous data. Continuous variables were presented as medians with interquartile ranges, whereas categorical data were summarized as frequencies and percentages. Median follow-up time was estimated using the reverse Kaplan–Meier analysis, calculated from diagnosis to last follow-up, with corresponding 95% confidence intervals (CIs). Group comparisons (non-VTE versus VTE) were performed to assess differences in baseline characteristics between the groups.

A time-dependent Cox proportional hazards model was used to evaluate the association between VTE and OS. VTE was implemented as a time-varying exposure variable, whereby patients contributed person-time as unexposed from the date of advanced LC diagnosis to the occurrence of the first VTE event and as exposed thereafter to death or censoring. VTE resolution was not modeled separately, and exposure status remained classified as VTE after the first event. Only the first VTE event was included in the time-dependent framework. KRS was treated as a baseline covariate measured at diagnosis and was not updated over time. Because KRS and VTE may overlap conceptually, we assessed the potential for collinearity when both variables were included in the survival model. OS was defined as the time from the date of LC diagnosis to either the date of death from any cause or the last follow-up. Results from the Cox model were reported as hazard ratios (HRs) with 95% CI. Kaplan–Meier analysis was used for descriptive estimation of OS and for graphical visualization of survival distributions.

For cumulative incidence analyses, VTE was treated as the outcome of interest with death considered a competing event. Cumulative incidence functions were estimated and plotted to describe the probability of developing VTE over time, accounting for the competing risk of death. Cumulative incidence functions were also stratified by KRS group status to evaluate the effect of KRS on the probability of developing VTE.

Competing risk regression was performed using the Fine-Gray subdistribution hazards model to identify predictors of VTE, with death included as a competing event. Univariable Fine-Gray regression analyses were conducted, with each variable evaluated separately rather than within a multivariable model, to avoid overfitting given the limited number of VTE events. Results were reported as subdistribution HR with 95% CI. As a sensitivity analysis, Fine-Gray regression was repeated after excluding patients with VTE events occurring before LC diagnosis (defined as within 90 d before diagnosis).

All statistical tests were two sided, and a *p* value of less than 0.05 was considered statistically significant. Statistical analyses were performed using R version 4.3.3 and IBM SPSS Statistics version 30.0.

## Results

### Baseline Patient and Tumor Characteristics

In our retrospective study, which encompassed 160 patients with advanced ALK-positive NSCLC, *ALK* fusion variants were identified in 97 subsequently enrolled patients. A total of 63 patients were excluded because they had incomplete data on the *ALK* fusion type. Table 1 summarizes the clinicopathologic characteristics and statistical analysis for both the VTE and non-VTE subgroups. Both groups were generally well balanced in terms of baseline patient and tumor characteristics. Notable differences were observed in laboratory parameters. Albumin levels were significantly lower in the VTE group, with 80.0% of patients having levels less than 3.5 g/L compared with 40.3% in the non-VTE group (*p* < 0.001). Leukocytosis (>11 × 10^9^/L) was also more frequent in the VTE group (48.6% versus 8.1%), as was thrombocytosis (34.2% versus 22.6%). Anemia (hemoglobin <100 g/L) occurred exclusively in the VTE group (11.4%). Furthermore, patients with VTE had higher incidences of skeletal (42.9% versus 38.7%), liver (31.4% versus 21.0%), and adrenal metastases (20.0% versus 8.1%), whereas brain metastases were more common in the non-VTE group (24.2% versus 17.1%). By the end of the study period, mortality was significantly higher among the patients with VTE (82.9% versus 48.4%, *p* < 0.001).

**Table 1:** Clinical and Demographic Characteristics of Patients With Advanced ALK-Positive NSCLC With and Without VTE Treated at Karolinska University Hospital Between January 2009 and December 2021

Variable	Level	Overall, n = 97	Non-VTE, n = 62	VTE, n = 35	*p*
Age at LC diagnosis (median [IQR])		60.0 [53.0, 66.0]	61.0 [52.3, 66.0]	58.0 [53.0, 65.0]	0.53
Age group (%)	<60	49 (50.5)	29 (46.8)	20 (57.1)	0.44
	>60	48 (49.5)	33 (53.2)	15 (42.9)	
Sex (%)	Female	57 (58.8)	39 (62.9)	18 (51.4)	0.38
	Male	40 (41.2)	23 (37.1)	17 (48.6)	
Smoking status (%)	Never smoker	61 (62.9)	41 (66.1)	20 (57.1)	0.67
	Former smoker	27 (27.8)	16 (25.8)	11 (31.4)	
	Smoker	9 (9.3)	5 (8.1)	4 (11.4)	
Histology (%)	Adenocarcinoma	93 (95.9)	60 (96.8)	33 (94.3)	0.47
	Adenosquamous carcinoma	1 (1.0)	0 (0.0)	1 (2.9)	
	Squamous cell carcinoma	2 (2.1)	1 (1.6)	1 (2.9)	
	Undifferentiated carcinoma	1 (1.0)	1 (1.6)	0 (0.0)	
ECOG (%)	0–1	92 (94.8)	58 (93.5)	34 (97.1)	0.77
	>2	5 (5.2)	4 (6.5)	1 (2.9)	
M stage (%)	M0	4 (4.1)	3 (4.8)	1 (2.9)	0.91
	M1a	20 (20.6)	13 (21.0)	7 (20.0)	
	M1b	30 (30.9)	20 (32.3)	10 (28.6)	
	M1c	43 (44.3)	26 (41.9)	17 (48.6)	
BMI (median [IQR])		24.10 [22.00, 26.40]	24.05 [21.92, 27.60]	24.30 [22.60, 25.65]	0.90
BMI group (%)	<22	24 (24.7)	16 (25.8)	8 (22.9)	0.84
	22–30	63 (64.9)	39 (62.9)	24 (68.6)	
	>30	10 (10.3)	7 (11.3)	3 (8.6)	
Albumin (%)	<35 g/L	53 (54.6)	25 (40.3)	28 (80.0)	**<0.001**
	≥35 g/L	44 (45.4)	37 (59.7)	7 (20.0)	
Leukocyte count (%)	>11x10^9^/L	15 (15.5)	4 (6.5)	11 (31.4)	**0.003**
	<11x10^9^/L	82 (84.5)	58 (93.5)	24 (68.6)	
Hemoglobin (%)	<100 g/L	3 (3.1)	0 (0.0)	3 (8.6)	0.08
	>100 g/L	94 (96.9)	62 (100.0)	32 (91.4)	
Thrombocyte count (%)	≥350x10^9^/L	21 (21.6)	14 (22.6)	7 (20.0)	1.00
	<350x10^9^/L	76 (78.4)	48 (77.4)	28 (80.0)	
Hypertension (%)	Yes	30 (30.9)	19 (30.6)	11 (31.4)	1.00
	No	67 (69.1)	43 (69.4)	24 (68.6)	
Diabetes (%)	Yes	5 (5.2)	5 (8.1)	0 (0.0)	0.21
	No	92 (94.8)	57 (91.9)	35 (100.0)	
Comorbidity (%)	≥1 comorbidity	44 (45.4)	29 (46.8)	15 (42.9)	0.87
	Previous healthy	53 (54.6)	33 (53.2)	20 (57.1)	
Brain metastasis (%)	Yes	21 (21.6)	15 (24.2)	6 (17.1)	0.58
	No	76 (78.4)	47 (75.8)	29 (82.9)	
Skeletal metastasis (%)	Yes	39 (40.2)	24 (38.7)	15 (42.9)	0.85
	No	58 (59.8)	38 (61.3)	20 (57.1)	
Liver metastasis (%)	Yes	24 (24.7)	13 (21.0)	11 (31.4)	0.37
	No	73 (75.3)	49 (79.0)	24 (68.6)	
Adrenal metastasis (%)	Yes	12 (12.4)	5 (8.1)	7 (20.0)	0.16
	No	85 (87.6)	57 (91.9)	28 (80.0)	
First-line treatment (%)	Chemotherapy	50 (51.5)	29 (46.8)	21 (60.0)	0.45
	No systemic treatment	3 (3.1)	2 (3.2)	1 (2.9)	
	Targeted therapy	44 (45.4)	31 (50.0)	13 (37.1)	
First-line ALK TKI (%)	Alectinib	27 (27.8)	20 (32.3)	7 (20.0)	0.28
	Ceritinib	3 (3.1)	3 (4.8)	0 (0.0)	
	Crizotinib	14 (14.4)	8 (12.9)	6 (17.1)	
ALK fusion variant (%)	1	38 (39.2)	21 (33.9)	17 (48.6)	0.26
	2	10 (10.3)	5 (8.1)	5 (14.3)	
	3a/b	31 (32.0)	23 (37.1)	8 (22.9)	
	Other variants	18 (18.6)	13 (21.0)	5 (14.3)	
KRS group (%)	Intermediate	91 (93.8)	61 (98.4)	30 (85.7)	**0.040**
	High	6 (6.2)	1 (1.6)	5 (14.3)	
Deceased (%)	Yes	59 (60.8)	30 (48.4)	29 (82.9)	**<0.001**
	No	38 (39.2)	32 (51.6)	6 (17.1)	

BMI, body mass index; ECOG, Eastern Cooperative Oncology Group; IQR, interquartile range; KRS, Khorana Risk Score; LC, lung cancer; TKI, tyrosine kinase inhibitor; VTE, venous thromboembolism.

Bold values indicate statistical significance (*p* < 0.05).

### Characteristics and Cumulative Incidence of Patients With Venous Thromboembolism

Among the 97 patients included in the study, 35 (36.1%) developed VTE during the follow-up. Most patients were classified in the intermediate KRS group (*n* = 91, 93.8%), whereas six patients (6.2%) were in the high-risk group; no patients fell into the low-risk category, as all had LC. PE accounted for most VTE events (68.6%), followed by DVT alone (17.1%), or a combination of both PE and DVT (14.3%). The median age at VTE diagnosis was 58 years (SD = 11.7), and 82.9% were symptomatic at clinical presentation. Most VTE events (62.9%) were diagnosed during an emergency department visit. The timing of VTE varied: 20.0% occurred within 90 days before the cancer diagnosis, 11.4% at diagnosis before treatment, 22.9% within the first 6 months of therapy, and 45.7% beyond 6 months after treatment initiation. At the time of VTE, 54.3% of patients were receiving an ALK TKI (most often crizotinib), 14.3% were receiving chemotherapy, and 31.4% had not yet initiated systemic therapy. Re-thrombosis occurred in 17.1% of patients. Only 2.9% of patients underwent surgery 30 days before their first VTE, whereas 20.0% were hospitalized within 30 days of the event (Table 2).

**Table 2:** Characteristics of Patients With ALK-Positive NSCLC With VTE

Characteristics	No. of Patients (%)
Total	35
Location of VTE	
Pulmonary	24 (68.6)
DVT	6 (17.1)
Both	5 (14.3)
Clinical presentation	
Asymptomatic/Incidental	6 (17.1)
Symptomatic	29 (82.9)
Patient situation	
Ambulatory	13 (37.1)
Emergency department	22 (62.9)
Time of occurrence	
Within 90 d before diagnosis	7 (20.0)
At diagnosis, before start of treatment	4 (11.4)
Within the first 6 mo of treatment	8 (22.9)
Beyond 6 mo from start of treatment	16 (45.7)
Cancer therapy at initial VTE	
No systemic treatment	11 (31.4)
Receiving chemotherapy	5 (14.3)
Receiving TKI	19 (54.3)
TKI treatment at initial VTE	
Crizotinib	12 (34.3)
Ceritinib	1 (2.9)
Alectinib	5 (14.3)
Re-thrombosis	
Yes	6 (17.1)
No	29 (82.9)
Surgery ≤ 30 d before first VTE	
Yes	1 (2.9)
No	34 (97.1)
Hospitalized ≤ 30 d before first VTE	
Yes	7 (20.0)
No	28 (80.0)
Days from diagnosis to first clot, median (IQR)	167.0 (14.5–579.5)

d, days; DVT, deep venous thrombosis; IQR, interquartile range; mo, months; TKI, tyrosine kinase inhibitor; VTE, venous thromboembolism.

**Figure 1:**
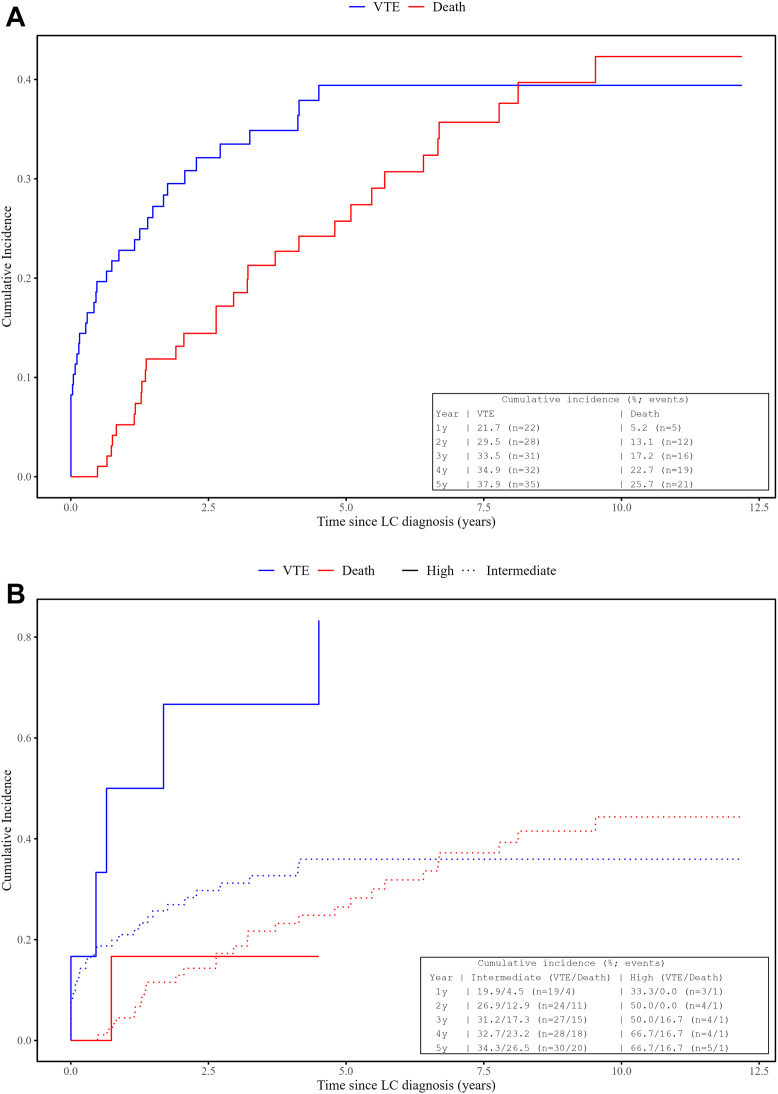
(*A*) Cumulative incidence of VTE and death from the time of diagnosis of advanced ALK-positive NSCLC. Estimated cumulative incidences at predefined yearly intervals are summarized in the inset table. (*B*) Cumulative incidence of VTE and death from the time of diagnosis of advanced ALK-positive NSCLC, stratified by high and intermediate Khorana risk score groups. Estimated cumulative incidences at predefined yearly intervals are summarized in the inset table. LC, lung cancer; VTE, venous thromboembolism.

**Supplementary Figure 1:**
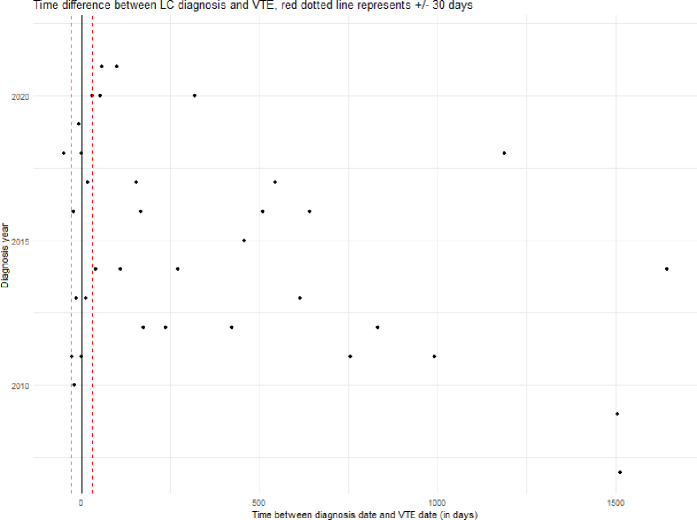
Time in days between the diagnosis of advanced ALK-positive non-small cell lung cancer and the occurrence of venous thromboembolism.

The cumulative incidence of VTE was highest in the first year after diagnosis (21.7%) and increased more gradually thereafter: 29.5% at year 2, 33.5% at year 3, 34.9% at year 4, and 37.9% at year 5 (Table 3A, Fig. 1*A*). Cumulative incidence was consistently higher in the high-risk KRS group compared with the intermediate-risk group throughout the follow-up (Table 3B, Fig. 1*B*). The distribution of time from LC diagnosis to VTE for the entire cohort is revealed in Supplementary Fig. 1; among patients who developed VTE, the median time to the first event was 167 days (interquartile range: 14.5–579.5).

**Table 3:** Cumulative Incidence of VTE and Death in the (A) First 5 Years After Lung Cancer Diagnosis and (B) Stratified by the KRS Groups.

Years	Outcome	Patients, *N*	Events, *N*	Rate per 100 Person-Year (95% CI)	Risk (%) (95% CI)
1	VTE	97	22	28.51 (17.87–43.17)	21.74 (15.48–31.96)
1	Death	97	5	6.48 (2.10–15.12)	5.23 (2.22–11.50)
2	VTE	97	28	21.07 (14.00–30.45)	29.52 (20.79–38.55)
2	Death	97	12	9.03 (4.67–15.77)	13.13 (7.22–20.39)
3	VTE	97	31	17.99 (12.22–25.54)	33.49 (23.52–41.77)
3	Death	97	16	9.29 (5.31–15.08)	17.17 (10.42–25.13)
4	VTE	97	32	15.68 (10.72–22.13)	34.86 (24.44–42.84)
4	Death	97	19	9.31 (5.60–14.54)	22.70 (12.91–28.58)
5	VTE	97	35	15.30 (10.66–21.28)	37.90 (27.22–46.00)
5	Death	97	21	9.18 (5.68–14.03)	25.73 (14.62–30.84)

KRS Group	Year	Outcome	Patients, N	Events, N	Rate per 100 Person-Year (95% CI)	Risk (%) (95% CI)
Intermediate	1	VTE	91	19	25.92 (15.60–40.47)	19.85 (13.79–30.32)
Intermediate	1	Death	91	4	5.46 (1.49–13.97)	4.47 (1.72–10.76)
High	1	VTE	6	3	78.02 (16.09–228.00)	33.33 (18.76–81.24)
High	1	Death	6	1	26.01 (0.66–144.89)	0.00 (3.01–56.35)
Intermediate	2	VTE	91	24	18.84 (12.07–28.04)	26.94 (18.41–36.25)
Intermediate	2	Death	91	11	8.64 (4.31–15.45)	12.91 (6.89–20.36)
High	2	VTE	6	4	72.34 (19.71–185.23)	50.00 (30.00–90.32)
High	2	Death	6	1	18.09 (0.46–100.77)	0.00 (3.01–56.35)
Intermediate	3	VTE	91	27	16.29 (10.73–23.70)	31.21 (21.26–39.72)
Intermediate	3	Death	91	15	9.05 (5.06–14.92)	17.26 (10.25–25.43)
High	3	VTE	6	4	61.26 (16.69–156.86)	50.00 (30.00–90.32)
High	3	Death	6	1	15.32 (0.39–85.33)	16.67 (3.01–56.35)
Intermediate	4	VTE	91	28	14.24 (9.46–20.58)	32.68 (22.23–40.87)
Intermediate	4	Death	91	18	9.16 (5.43–14.47)	23.20 (12.89–29.11)
High	4	VTE	6	4	53.13 (14.48–136.03)	66.67 (30.00–90.32)
High	4	Death	6	1	13.28 (0.34–74.00)	16.67 (3.01–56.35)
Intermediate	5	VTE	91	30	13.59 (9.17–19.40)	34.31 (24.17–43.14)
Intermediate	5	Death	91	20	9.06 (5.54–14.00)	26.47 (14.70–31.52)
High	5	VTE	6	5	62.24 (20.21–145.25)	66.67 (43.65–96.99)
High	5	Death	6	1	12.45 (0.32–69.36)	16.67 (3.01–56.35)

CI, confidence interval; KRS, Khorana Risk Score; VTE, venous thromboembolism.

**Table 4:** Univariable Fine and Gray’s Competing Risk Regression Analysis Expressed as sHR for the Occurrence of Venous Thromboembolism and Death in Patients With ALK-Positive NSCLC

Variable	Level	Fine and Gray’s Competing Risk Regression				
		*N*	sHR	95% CI Lower	95% CI Upper	*p* Value
Age		97	1	0.97	1.02	0.78
Age group	≤60	49	Ref.			
	>60	48	0.71	0.37	1.37	0.31
Sex	Female	57	Ref.			
	Male	40	1.39	0.72	2.65	0.32
Smoking status	Never smoker	61	Ref.			
	Former smoker	27	1.24	0.61	2.51	0.56
	Smoker	9	1.49	0.51	4.35	0.47
ECOG	0–1	92	Ref.			
	>2	5	0.52	0.06	4.34	0.54
M-stage	M0	4	Ref.			
	M1a	20	1.18	0.15	9.51	0.87
	M1b	30	1.33	0.17	10.54	0.79
	M1c	43	1.65	0.22	12.53	0.63
BMI		97	1	0.92	1.09	0.93
BMI group	<25	58	Ref.			
	≥25	39	1.24	0.65	2.37	0.52
Leukocyte count >11 x 10^9^/L	No	82	Ref.			
	Yes	15	3.74	1.91	7.31	**<0.001**
Hemoglobin <100 g/L	No	94	Ref.			
	Yes	3	5.18	2.07	12.94	**<0.001**
Thrombocyte count ≥350×10⁹/L	Yes	22	Ref.			
	No	75	1.0	0.36	1.34	0.99
Albumin ≥35 g/L	No	53	Ref.			
	Yes	44	0.24	0.10	0.56	**<0.001**
Hypertension	No	67	Ref.			
	Yes	30	1.08	0.53	2.17	0.84
Diabetes	No	92	Ref.			
	Yes	5	0	0	0	0
Comorbidity	≥1 comorbidity	44	Ref.			
	Previous healthy	53	1.13	0.58	2.19	0.72
Brain metastasis	Yes	21	Ref.			
	No	76	1.39	0.61	3.21	0.44
Skeletal metastasis	Yes	39	Ref.			
	No	58	0.89	0.46	1.71	0.72
Liver metastasis	Yes	24	Ref.			
	No	73	0.65	0.32	1.3	0.22
Adrenal metastasis	No	85	Ref.			
	Yes	12	2.92	1.19	7.16	**0.019**
First-line treatment	Chemotherapy	50	Ref.			
	No systemic treatment	3	0.92	0.10	8.33	0.94
	Targeted therapy	44	0.82	0.42	1.61	0.56
First-line ALK TKI	Alectinib	27	Ref.			
	Ceritinib	3	0	0	0	0
	Crizotinib	14	1.67	0.57	4.86	0.35
ALK fusion variant	1	38	Ref.			
	2	10	1.04	0.39	2.82	0.93
	3a/b	31	0.5	0.21	1.18	0.11
	Other variants	18	0.5	0.19	1.28	0.15
KRS group	Intermediate	91	Ref.			
	High	6	3.12	1.49	6.53	**<0.001**

BMI, body mass index; CI, confidence interval; ECOG, Eastern Cooperative Oncology Group; KRS, Khorana Risk Score; Ref., reference; sHR, subdistributed hazard ratio; TKI, tyrosine kinase inhibitor.

Bold values indicate statistical significance (*p* < 0.05).

### Time-Dependent Cox Proportional Hazards Model to Evaluate the Impact of Venous Thromboembolism on Survival Outcomes

The median follow-up time in our study was 94.0 months (95% CI: 54.1–133.9). The results of the time-dependent Cox proportional hazards model, with and without VTE, among patients with ALK-positive NSCLC are presented in Supplementary Table 1, along with an additional analysis stratified by the KRS group.

In model 1, we evaluated whether VTE occurrence was associated with mortality, and in model 2, we evaluated the impact of both VTE and KRS groups on mortality. The presence of VTE was associated with a significantly increased hazard of mortality in patients with ALK-positive NSCLC (HR = 3.43, 95% CI: 2.15–5.46, *p* < 0.001). In model 2, when considering KRS as a stratification variable, we found a HR of 3.09 (95% CI: 1.92–4.99, *p* value < 0.001) for the VTE group and HR of 1.81 (95% CI: 1.01–3.24, *p* value = 0.04) for the high KRS group compared with the intermediate group. No meaningful collinearity between KRS and VTE was observed.

### Survival Outcomes in the Context of Venous Thromboembolism

Among the 35 patients who developed VTE, 15 died within 6 months of the event, including seven within the first month and two on the day of VTE diagnosis; all were classified as cancer-related deaths. Patients who developed VTE had substantially shorter survival compared with the overall cohort. The median survival for patients with VTE was 25.0 months (95% CI: 17.0–33.0), and survival measured from the time of VTE diagnosis was 13.0 months (95% CI: 6.7–19.3). In contrast, the median OS among patients without VTE was 76.0 months (95% CI: 51.7–100.3) (Supplementary Table 3, Supplementary Fig. 2).

### Risk Factors for the Development of Venous Thromboembolism

Risk factors associated with VTE in the competing risk regression analysis are summarized in Table 4. In the competing risk regression analysis for VTE, several variables were significantly associated with an increased risk: high KRS (HR = 3.12, 95% CI: 1.49–6.53), compared with the intermediate-risk group; leukocyte count more than 11 × 10^9^/L (HR = 3.74, 95% CI: 1.91–7.31), compared with less than or equal to 11 × 10^9^/L; hemoglobin less than 10 g/dL (HR = 5.18, 95% CI: 2.07–12.94), compared with more than or equal to 10 g/dL; and the presence of adrenal metastasis (HR = 2.92, 95% CI: 1.19–7.16), compared with absence of the adrenal metastasis. Conversely, albumin levels more than or equal to 3.5 g/dL were associated with a reduced risk of VTE (HR = 0.24, 95% CI: 0.10–0.56), compared with levels less than 3.5 g/dL.

In a sensitivity analysis excluding patients (*n* = 7) who had VTE within 90 days before their LC diagnosis, the risk factor associations remained consistent with the primary competing risk regression. In particular, leukocytosis (>11 × 10^9^/L), low hemoglobin (<10 g/dL), and high KRS continued to reveal elevated subdistributed HR, indicating that prediagnosis VTE events did not substantially influence the overall findings (Supplementary Table 2).

## Discussion

This retrospective study provides valuable insights into the impact of VTE on the clinical outcomes of patients with advanced ALK-positive NSCLC. It highlights VTE as a prevalent and critical complication that serves as an independent prognostic marker significantly influencing survival. The strength of our study is a long follow-up period of 94 months, which provides long-term data on VTE incidence and survival outcomes. We also used competing risk regression and time-dependent Cox proportional hazards models, which account for biases such as competing risks (e.g., death), thereby improving the reliability of the analyses.

We found that, after adjusting for time, patients with ALK-positive NSCLC with VTE have 3.43 times the risk of death compared with those without VTE. This elevated mortality risk may reflect VTE as a surrogate marker of a more aggressive tumor biology and poorer prognosis. These results highlight the clinical importance of incorporating VTE risk assessment and proactive management into the standard care of this patient population. Similar observations have been reported in previous studies: Zer et al.^8^ found a significantly worse outcome in patients with VTE, with an HR of 5.71, whereas another study identified thromboembolic disease as an independent predictor of OS (HR = 1.70).^31^

The poorer outcome associated with VTE is notable given that ALK-positive patients are typically younger and often have fewer traditional cardiovascular risk factors. In our cohort, age and comorbidity burden were similar between VTE and non-VTE groups, suggesting that these factors alone are unlikely to explain the observed differences in survival. This supports prior observations that VTE in ALK-rearranged NSCLC may reflect underlying tumor biology rather than patient-related risk factors.^32^

The incidence of VTE was 36.7% in our cohort, markedly higher than the approximately 6% reported in the general LC population,^33^ yet consistent with previous studies in ALK-positive NSCLC reporting rates between 26.9% and 47.1%.^8^^,^^31^^,^^34^^,^^35^ This high incidence highlights the need for vigilant screening and early intervention to manage VTE in these patients. Our findings reveal that VTE risk in ALK-rearranged NSCLC is highest during the first year after diagnosis, followed by a lower incidence in subsequent years.

The mechanisms behind the increased risk of thrombosis in patients with cancer are still far from clear but relate to both tumor-, treatment-, and patient-related factors. It could be factors such as high age, ethnicity, comorbidities, histologic tumor subtype, vascular compression, vessel damage, use of central catheters, chemotherapy, and hypercoagulability of the blood. The latter may be triggered by the activation of cancer-associated procoagulant pathways or by the indirect systemic effects of cancer on several normal cell types, including leukocytes and endothelial cells.^36^ The high incidence of VTE in patients with ALK-positive NSCLC may be partly explained by the histopathologic characteristics of these tumors, which often exhibit a mucinous cribriform pattern.^11^^,^^37^ Mucin production has been linked to thromboembolism in patients with lung adenocarcinoma^38^ and could be explained by a hypothesis that mucin promotes procoagulant secretion, leading to platelet activation and the subsequent formation of microthrombi within the microvasculature.^39^ Furthermore, it has been suggested that ALK fusion proteins may activate STAT3 signaling and NLRP3 inflammasomes in macrophages, leading to increased inflammation, which is believed to contribute to a prothrombotic state.^40^^,^^41^ In the competing risks regression analysis, we found that adrenal metastasis was associated with an increased risk of VTE, suggesting that metastatic location may play a key role in thrombotic events.

Moreover, leukocytosis and anemia were significant predictors of VTE in our study, suggesting a putative complex interplay between systemic inflammation and anemia in the pathogenesis of thrombosis. We also found that a high KRS more than or equal to 3 points increased the risk, but this finding should be interpreted cautiously because it was based on very small patient numbers, which limits statistical robustness. Therefore, this finding should be considered exploratory and hypothesis generating rather than a definitive clinical risk factor. The interpretation of the KRS association is further constrained by the overlap with individual laboratory variables, emphasizing the need to validate our findings in larger ALK-positive NSCLC cohorts. We found that high albumin levels were associated with a reduced risk of VTE, potentially indicating that patients' better nutritional status confers some protection against thrombosis.

One study found an increased risk of VTE during TKI therapy, particularly with crizotinib (HR = 8.72) and alectinib (HR = 21.47), and identified liver metastases and baseline leukocytosis as risk factors. Notably, unlike our findings, that study reported that high KRS predicted shorter OS but not VTE.^42^ Another study found no significant VTE predictors apart from race, but it is likely attributable to a limited sample size of the study cohort.^8^

A meta-analysis also demonstrated that crizotinib was associated with a higher risk of thromboembolism than chemotherapy and newer-generation ALK TKIs, with ceritinib demonstrating the lowest risk.^43^ Consistently, most VTE events in our cohort occurred during crizotinib treatment.

The clinical characteristics of the ALK-positive patients in our cohort were similar to those reported in previous studies, including younger, mainly never-smoking patients with an even sex distribution ratio.^44^

Approximately 31.4% of patients developed VTE at the time of NSCLC diagnosis, whereas the remaining patients developed it subsequently. The most common site of VTE was pulmonary, with most patients presenting with symptoms. Notably, very few patients had surgery or hospitalization within 1 month before VTE onset, despite these being well-known risk factors. We found that *ALK* fusion variant 3a/b and variant 2, compared with variant 1, had a lower risk of developing VTE, but this was not statistically significant, suggesting that further investigation in larger cohorts is warranted to fully elucidate the role of specific *ALK* fusion variants in the thrombotic process. The main difference between the most common *ALK* variants is a TAPE protein domain that affects protein stability. The “longer” *ALK* variants, such as variants 1 and 2, result in an unstable EML4–ALK protein, whereas variants 3a/b and 5a/b, which lack a TAPE domain, are short and stable.^45^ One study revealed that *EML4–ALK* fusion variant 1 activates H-, N-, and K-RAS to drive mitogen-activated protein kinase (MAPK) signaling via the hydrophobic motif in EML proteins (HELP) within the TAPE domain.^46^ Such increased MAPK activation could play an essential role in platelet activation and thus promote thrombus formation.^47^ A study by Su et al.^48^ demonstrated that the EML4-ALK fusion protein may increase venous thrombogenicity by modulating the expression of the coagulation factor TF. The study suggested that this process could involve the ERK1/2 signaling pathway, which activates the transcription factor AP-1. In turn, AP-1 induces the up-regulation of TF expression, thereby initiating the blood coagulation cascade and contributing to the cancer-associated VTE.

Several limitations of our study need to be considered. Our results were obtained in a retrospective study design in a single-center institution. The number of included patients was limited due to the rarity of EML4–ALK-positive NSCLC with known fusion variants. Moreover, the lack of reflex testing for *EML4*–*ALK* fusion variants before 2015 at our institution meant patients were enrolled only if sufficient tumor specimens were available for RT-PCR or NGS analysis. The RT-PCR analysis setup meant that all *ALK* fusion variants could not be specified, and *EML4*–*ALK* variants 4 to 8 were categorized as other variants. Thus, further studies are required to determine the impact of fusion variants on the development of VTE in patients with ALK-positive NSCLC. Furthermore, the study period spanned from 2009 to 2021, during which treatment paradigms evolved significantly. A considerable number of patients received upfront chemotherapy, including intravenous regimens and central venous access devices, which are no longer part of standard first-line treatment for ALK-positive NSCLC. This temporal heterogeneity may have introduced confounding related to treatment-associated thrombosis risk. We also chose not to focus on arterial thromboembolism because previous studies have not revealed an increased risk in this patient group.^32^^,^^49^ Although we attempted to account for death as a potential confounder in our competitive risk regression analyses, residual confounding remains a possibility because we did not evaluate all risk factors for VTE. Factors such as trauma, prolonged immobilization, long-distance travel, pregnancy, or central venous catheter use were not collected because they are uncommon in ALK-positive NSCLC and were inconsistently documented in our retrospective records, limiting their reliable extraction.

Cause-of-death information was available for all deceased patients and was attributed in every case to progressive NSCLC, with no deaths documented as VTE related. Although we cannot completely exclude the possibility that unrecognized VTE contributed to individual cases, particularly given that fatal PE may be underdiagnosed in advanced cancer, the available documentation suggests that mortality was primarily driven by disease progression. At the same time, descriptive survival data from our cohort reveal that patients who developed VTE had shorter OS than those who did not. Together, these limitations highlight the need for larger prospective studies with standardized data collection to more accurately evaluate thrombotic events in advanced ALK-positive NSCLC.

## Conclusions

In conclusion, this study emphasizes the urgent need for early detection and proactive management of VTE in patients with ALK-positive NSCLC. It is essential for physicians to actively assess for signs and symptoms of thrombosis at diagnosis and throughout the follow-up period to rule out thromboembolic disease. Enhanced surveillance strategies may need to be considered for patients with elevated risk, particularly those with baseline adrenal metastasis, leukocyte count more than 11 × 10^9^/L, and hemoglobin less than 10 g/dL. However, several of these findings were based on small patient numbers and should be interpreted as exploratory. Validation in larger cohorts is needed before these factors can be considered clinically meaningful. The significant influence of VTE on OS suggests that integrating systematic VTE risk assessment into routine clinical practice could substantially improve patient outcomes. Although our findings do not support the routine use of prophylactic anticoagulation, the risks and benefits of prophylactic interventions can be considered on an individual basis. Future studies with larger, prospectively followed cohorts are needed to clarify whether prophylactic anticoagulation has a meaningful role in reducing VTE incidence in patients with ALK-positive NSCLC. Incorporating tools such as the KRS may also help clinicians identify patients at increased risk, thereby guiding more targeted and effective preventive strategies.

## CRediT Authorship Contribution Statement

**Caroline Kamali**: Conceptualization, Methodology, Investigation, Data curation, Formal analysis, Data interpretation, Visualization, Writing – original draft, Writing – review & editing.

**Georgios Tsakonas**: Methodology, Writing – review & editing.

**Rolf Lewensohn**: Resources, Funding acquisition, Writing – review & editing.

**Lena Kanter**: Methodology.

**Hanna Vikman**: Formal analysis, Methodology, Data interpretation, Writing – review & editing.

**Anders Berglund**: Formal analysis, Methodology, Data interpretation, Validation.

**Elena Nedbaylo**: Investigation.

**Per Hydbring**: Writing – review & editing.

**Luigi De Petris**: Supervision, Writing – review & editing.

**Kristina Viktorsson**: Resources, Funding acquisition, Writing – review & editing.

**Simon Ekman**: Conceptualization, Supervision, Resources, Funding acquisition, Project administration, Writing – original draft, Writing – review & editing.

## Disclosure

The authors declare no conflict of interest.

## Data Availability Statement

The data supporting the findings of this study are not publicly accessible to protect the privacy of the study participants. However, these data may be made available on reasonable request to the corresponding author.

## Footnotes

Note: To access the supplementary material accompanying this article, visit the online version of the *JTO Clinical and Research Reports* at www.jtocrr.org and at https://doi.org/10.1016/j.jtocrr.2026.101003.
