# Supplementary material for: Risk Factors for Developing Venous Thromboembolism in Patients With Advanced ALK-Rearranged NSCLC
Source: JTO Clin Res Rep. 2026 Apr 23;7(6):101003. doi: 10.1016/j.jtocrr.2026.101003 (PMC13226909; doi:10.1016/j.jtocrr.2026.101003)
Supplement: Supplementary Table 1 [file mmc1.docx]

| **Supplementary Table 1.** Time-dependent Cox proportional hazards models to evaluate the impact of venous thromboembolism (VTE) on survival outcomes among patients with ALK-positive NSCLC, with an additional analysis stratified by the KRS group. |
| --- |

| **Model 1** | **Term** | **HR** | **95% CI** | **Standard Error** | **Z Statistic** | **P-Value** |
| --- | --- | --- | --- | --- | --- | --- |
| VTE | VTE | 3.43 | 2.15-5.46 | 0.24 | 5.19 | <0.001 |
|  | Non-VTE | Ref |  |  |  |  |
| **Model 2** | **Term** | **HR** |  | **Standard Error** | **Z Statistic** | **P-Value** |
| VTE with KRS | VTE | 3.08 | 1.89-5.0 | 0.25 | 4.55 | <0.001 |
|  | Non-VTE | Ref |  |  |  |  |
|  | KRS High | 1.93 | 0.99-3.77 | 0.34 | 1.94 | 0.05 |
|  | KRS Intermediate | Ref |  |  |  |  |
