# Supplementary material for: Risk Factors for Developing Venous Thromboembolism in Patients With Advanced ALK-Rearranged NSCLC
Source: JTO Clin Res Rep. 2026 Apr 23;7(6):101003. doi: 10.1016/j.jtocrr.2026.101003 (PMC13226909; doi:10.1016/j.jtocrr.2026.101003)
Supplement: Supplementary Table 2 [file mmc2.docx]

**Supplementary Table 2.** Univariable Fine and Gray’s Competing risk regression analysis expressed as subdistributed hazard ratios (sHR) for the occurrence of venous thromboembolism and death in patients with ALK-positive non-small cell lung cancer, with sensitivity analysis excluding pre-diagnosis VTE events.

|  |  | **Fine and Gray’s Competing risk regression** | | | | |
| --- | --- | --- | --- | --- | --- | --- |
| **Variable** | **Level** | **N** | **sHR** | **HR_lower** | **HR_upper** | **P-value** |
| Age |  | 90 | 1 | 0.97 | 1.02 | 0.75 |
| Age group | ≤60 | 44 | Ref. |  |  |  |
|  | >60 | 46 | 0.8 | 0.38 | 1.68 | 0.56 |
| Sex | Female | 53 | Ref. |  |  |  |
|  | Male | 37 | 1.48 | 0.71 | 3.08 | 0.29 |
| Smoking status | Never smoker | 57 | Ref. |  |  |  |
|  | Former smoker | 25 | 1.26 | 0.57 | 2.78 | 0.56 |
|  | Smoker | 8 | 1.43 | 0.41 | 4.95 | 0.57 |
| ECOG | 0-1 | 85 | Ref. |  |  |  |
|  | >=2 | 5 | 0.64 | 0.07 | 5.86 | 0.7 |
| M-stage | M0 | 4 | Ref. |  |  |  |
|  | M1a | 19 | 0.98 | 0.11 | 8.56 | 0.98 |
|  | M1b | 28 | 1.06 | 0.12 | 9.32 | 0.96 |
|  | M1c | 39 | 1.28 | 0.15 | 10.63 | 0.82 |
| BMI |  | 90 | 1.03 | 0.93 | 1.13 | 0.61 |
| BMI group | <25 | 52 | Ref. |  |  |  |
|  | >=25 | 38 | 1.69 | 0.81 | 3.52 | 0.16 |
| Leukocyte >11 | No | 77 | Ref. |  |  |  |
|  | Yes | 13 | 4.34 | 2.02 | 9.32 | **<0.001** |
| hemoglobin <100 | No | 88 | Ref. |  |  |  |
|  | Yes | 2 | 5.26 | 1.77 | 15.64 | **<0.001** |
| TPK ≥350 | Yes | 20 | Ref. |  |  |  |
|  | No | 70 | 1.08 | 0.46 | 2.58 | 0.86 |
| Albumin | <35 | 48 | Ref. |  |  |  |
|  | ≥35 | 42 | 0.2 | 0.07 | 0.53 | **<0.001** |
| Hypertension | No | 63 | Ref. |  |  |  |
|  | Yes | 27 | 0.95 | 0.43 | 2.14 | 0.91 |
| Diabetes | No | 85 | Ref. |  |  |  |
|  | Yes | 5 | 0 | 0 | 0 | 0 |
| Comorbidity | ≥1 comorbidity | 41 | Ref. |  |  |  |
|  | Previous healthy | 49 | 1.14 | 0.54 | 2.39 | 0.74 |
| Brain metastasis | Yes | 21 | Ref. |  |  |  |
|  | No | 69 | 1.07 | 0.44 | 2.58 | 0.88 |
| Skeletal metastasis | Yes | 35 | Ref. |  |  |  |
|  | No | 55 | 1.02 | 0.48 | 2.17 | 0.95 |
| Liver metastasis | Yes | 21 | Ref. |  |  |  |
|  | No | 69 | 0.72 | 0.32 | 1.61 | 0.42 |
| Adrenal metastasis | No | 80 | Ref. |  |  |  |
|  | Yes | 10 | 2.97 | 0.99 | 8.87 | 0.052 |
| First-line treatment | Chemotherapy | 47 | Ref. |  |  |  |
|  | No systemic treatment | 3 | 1.11 | 0.11 | 11.77 | 0.93 |
|  | Targeted therapy | 40 | 0.69 | 0.31 | 1.52 | 0.35 |
| First-line ALK-TKI | Alectinib | 25 | Ref. |  |  |  |
|  | Ceritinib | 3 | 0 | 0 | 0 | 0 |
|  | Crizotinib | 12 | 1.57 | 0.41 | 5.99 | 0.51 |
| *ALK*-fusion variant | 1 | 35 | Ref. |  |  |  |
|  | 2 | 9 | 1 | 0.32 | 3.08 | 0.99 |
|  | 3a/b | 29 | 0.44 | 0.17 | 1.17 | 0.1 |
|  | Other variants | 17 | 0.45 | 0.16 | 1.3 | 0.14 |
| KRS group | Intermediate | 85 | Ref. |  |  |  |
|  | High | 5 | 3.32 | 1.47 | 7.52 | **<0.001** |
