# Supplementary material for: Risk Factors for Developing Venous Thromboembolism in Patients With Advanced ALK-Rearranged NSCLC
Source: JTO Clin Res Rep. 2026 Apr 23;7(6):101003. doi: 10.1016/j.jtocrr.2026.101003 (PMC13226909; doi:10.1016/j.jtocrr.2026.101003)
Supplement: Supplementary Table 3 [file mmc3.docx]

**Supplementary Table 3.** Survival outcomes in patients with and without venous thromboembolism.

| **Outcome** | **Value** |
| --- | --- |
| Patients with VTE (n) | 35 |
| Deaths within 6 months after VTE, n (%) | 15 (42.9%) |
| Deaths within 1 month after VTE, n (%) | 7 (20.0%) |
| Deaths on same day as VTE diagnosis, n (%) | 2 (5.7%) |
| Median OS in VTE group (months, 95% CI) | 25.0 (17.0–33.0) |
| Median OS from VTE diagnosis (months, 95% CI) | 13.0 (6.7–19.3) |
| Median OS in non-VTE group (months, 95% CI) | 76.0 (51.7-100.3) |
| Median OS in entire cohort (months, 95% CI) | 43.0 (28.7–57.3) |

CI, confidence interval; OS, overall survival.
