# Supplementary figures and images for: Risk Factors for Developing Venous Thromboembolism in Patients With Advanced ALK-Rearranged NSCLC
Source: JTO Clin Res Rep. 2026 Apr 23;7(6):101003. doi: 10.1016/j.jtocrr.2026.101003 (PMC13226909; doi:10.1016/j.jtocrr.2026.101003)

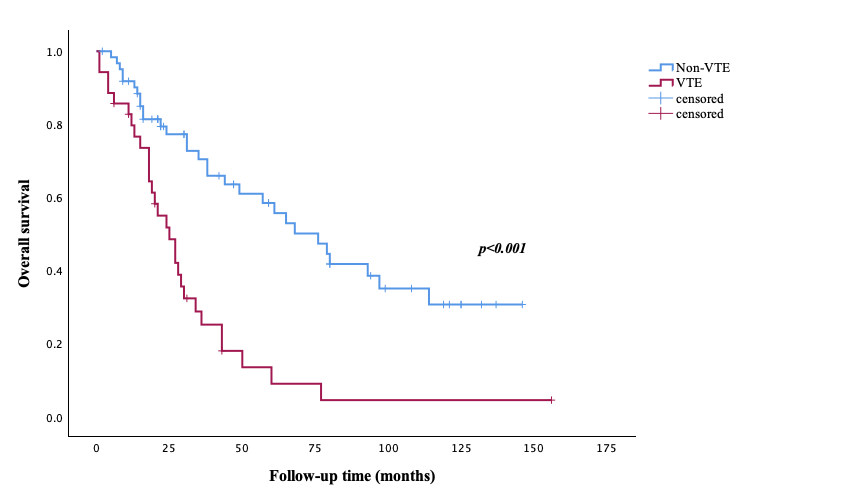


| **Non-VTE** | 62 | 36 | 24 | 18 | 9 | 5 | 0 | 0 |
| --- | --- | --- | --- | --- | --- | --- | --- | --- |
| **VTE** | 35 | 16 | 4 | 2 | 1 | 1 | 1 | 0 |

Supplement: Supplementary figure 1 [file mmc4.docx]
